# Superando a Montanha-Russa Hormonal ao Longo da Vida das Mulheres: Um Ponto de Virada para a Prevenção Cardiovascular

**DOI:** 10.36660/abc.20220259

**Published:** 2022-05-04

**Authors:** M. Julia Machline-Carrion

**Affiliations:** 1 EpHealth Primary Care Solutions São Paulo SP Brasil EpHealth Primary Care Solutions, São Paulo, SP – Brasil

**Keywords:** Terapia de Reposição de Estrogênios/efeitos adversos, Mulheres, Menopausa, Progesterona/efeitos adversos, Prevenção e Controle, Fatores de Risco

Embora as doenças cardiovasculares (DCV), especialmente a doença arterial coronariana e o acidente vascular cerebral, sejam a principal causa de morte e incapacidade no Brasil, tanto em mulheres quanto em homens,^1 , 2^ a prevenção cardiovascular (CV) implica uma abordagem abrangente das diferenças inerentes ao sexo. Nesse sentido, compreender, por exemplo, o papel da hipertensão, importante fator de risco para doença arterial coronariana e acidente vascular cerebral em mulheres brasileiras,^3^ no contexto das especificidades dessa população é fundamental.^1^

Distúrbios hipertensivos, incluindo hipertensão induzida pela gravidez (ocorrendo em 6-7% das gestações) e pré-eclâmpsia/eclâmpsia (ocorrendo em até 10% das gestações), são importantes fatores de risco CV^4 , 5^ que devem ser considerados ao avaliar o risco CV das mulheres.^1^

À medida que as mulheres envelhecem e os níveis de estrogênio diminuem, aumentam os riscos de osteoporose e doenças cardiovasculares.^6^ Os sintomas vasomotores (ondas de calor e suores noturnos), prevalentes entre mulheres na perimenopausa tardia e na menopausa recente, estão associados a um risco aumentado de doenças cardiovasculares e alterações cognitivas.^7 , 8^ Embora a terapia hormonal da menopausa (THM) continue sendo o tratamento mais eficaz para os sintomas vasomotores da menopausa,^8^ sua associação com hipertensão permanece incerta.^9 - 13^ Estudos observacionais sugeriram anteriormente riscos reduzidos de doença cardiovascular e demência com terapia hormonal pós-menopausa,^9^ mas a publicação inicial em 2002 dos resultados de um estudo randomizado e controlado conduzido pela Women’s Health Initiative (WHI) relatou riscos aumentados de doença cardiovascular, tromboembolismo venoso (TEV) e câncer de mama.^10^ O tratamento de ambos os grupos no estudo WHI foi interrompido precocemente para evitar possíveis danos. Em comparação com placebo, a terapia combinada (0,625 mg de estrogênios equinos conjugados [CEE] mais 2,5 mg de acetato de medroxiprogesterona) aumentou o risco anual de DAC em 0,6 por mil mulheres e de acidente vascular cerebral e câncer de mama em 0,9 por mil mulheres. Análises post hoc subsequentes considerando a idade e o tempo desde o início da menopausa (com menopausa definida como 12 meses sem menstruação) sugeriram riscos aumentados de doença coronariana e acidente vascular cerebral entre participantes do WHI que iniciaram a terapia hormonal após os 60 anos de idade, apoiando assim a “hipótese do tempo”.^10^ Na coorte *Etude Épidémiologique de femmes de la Mutuelle Générale de l’Education* (E3N), a MHT foi associada a um aumento modesto, mas significativo, do risco de hipertensão, especialmente ao usar estrogênio oral em combinação com um progestagênio, como derivados de pregnano e norpregnano.^14^

Em um estudo transversal da avaliação inicial do estudo ELSA-Brasil, incluindo 2.138 mulheres que passaram pela menopausa natural, Ferreira-Campos et al.,^15^ avaliaram a relação entre THM e hipertensão (definida como PA ≥140/90 mmHg ou uso prévio de algum anti-hipertensivo). Os autores constataram que 1.492 mulheres (69,8%) nunca usaram TH, 457 (21,4%) eram usuárias anteriores e 189 (8,8%) eram usuárias atuais. Neste estudo, as usuárias atuais de MHT foram menos propensas a apresentar hipertensão do que as mulheres que nunca usaram MHT (Odds Ratio [OR]=0,59; IC 95% 0,41-0,85). Além disso, as usuárias atuais de MTH apresentaram pressão arterial sistólica mediana menor do que as mulheres que nunca usaram MTH e usuárias anteriores (113 mmHg, 118,5 mmHg e 120 mmHg, respectivamente, (p=0,001).^15^ As conclusões deste estudo, embora cautelosas, contrastam com as recentes avaliações longitudinais maiores de estudos de coorte e ensaios clínicos. Nesse caso, a natureza transversal do estudo pode representar uma limitação. A “hipótese do tempo” não possível de avaliar neste desenho de estudo pode ser um determinante para um resultado diferente. Embora as análises transversais constituam um corpo substancial de geradores de evidências, as avaliações longitudinais e a “mágica da randomização” podem ser informativas para certas questões de pesquisa.
