# Supplementary material for: Superando a Montanha-Russa Hormonal ao Longo da Vida das Mulheres: Um Ponto de Virada para a Prevenção Cardiovascular
Source: Arq Bras Cardiol. 2022 May 4;118(5):914–5. [Article in Portuguese] doi: 10.36660/abc.20220259 (PMC9368890; doi:10.36660/abc.20220259)
Supplement: Translation

# Translation: Overcoming Women’s Lifelong Hormonal Rollercoaster: A Turning Point for Cardiovascular Prevention

**Authors:** M. Julia Machline-Carrion

**Affiliations:** 1 Brazil EpHealth Primary Care Solutions, São Paulo, SP – Brazil

**Keywords:** Estrogen Replacement Therapy/adverse effects, Women, Menopause, Progestins/adverse effects, Prevention and Control, Risk Factors

Although cardiovascular diseases (CVD), especially coronary artery disease and stroke, are the leading cause of death and disability in Brazil, both in women and men,^1 , 2^ addressing cardiovascular (CV) prevention implies a comprehensive approach to the inherent differences related to sex. In this sense, understanding, for instance, the role of hypertension, a major risk factor for coronary artery disease and stroke in Brazilian women,^3^ in the context of this population’s specificities is key.^1^

Hypertensive disorders, including pregnancy-induced hypertension (occurring in 6-7% of pregnancies) and preeclampsia/eclampsia (occurring in up to 10% of pregnancies), are important CV risk factors^4 , 5^ that should be accounted for when assessing women’s CV risk.^1^

As women age and estrogen levels decline, risks increase for osteoporosis and cardiovascular disease.^6^ Vasomotor symptoms (hot flashes and night sweats), prevalent among late perimenopausal and recently menopausal women, are linked to an increased risk of cardiovascular disease and cognitive changes.^7 , 8^ Although menopausal hormone therapy (MHT) remains the most effective treatment for vasomotor symptoms of menopause,^8^ its association with hypertension remains unclear.^9 - 13^ Observational studies have previously suggested reduced risks of cardiovascular disease and dementia with postmenopausal hormone therapy,^9^ but the initial publication in 2002 of findings from a randomized, controlled trial conducted by the Women’s Health Initiative (WHI) reported increased risks of cardiovascular disease, venous thromboembolism (VTE), and breast cancer.^10^ Treatment of both groups in the WHI trial was stopped early to prevent possible harm. Compared with placebo, combination therapy (0.625 mg of conjugated equine estrogens [CEE] plus 2.5 mg of medroxyprogesterone acetate) increased the annual risk of CVD by 0.6 per one thousand women and of stroke and breast cancer by 0.9 per one thousand women. Subsequent post hoc analyses conducted according to age and time from the onset of menopause (with menopause defined as 12 months without a menstrual period) suggested increased risks of coronary heart disease and stroke among WHI participants who started hormone therapy after the age of 60 years, thus supporting the “timing hypothesis”.^10^ In the Etude *Épidémiologique de femmes de la Mutuelle Générale de l’Education* (E3N) cohort, MHT was associated with a modest but significant increased risk of incident hypertension, especially when using oral estrogen in combination with a progestogen such as pregnane and norpregnane derivatives.^14^

In a cross-sectional study from the baseline assessment of the ELSA-Brazil study, including 2.138 women that have undergone natural menopause, Ferreira-Campos et al.^15^ assessed the relation between MHT and hypertension (defined as BP ≥140/90 mmHg or previous use of any antihypertensive drugs). The authors have found that 1.492 women (69.8%) have never used MHT, 457 (21.4%) were previous users, and 189 (8.8%) were current users. In this study, current MHT users were less likely to present hypertension than women who have never used MHT (Odds Ratio [OR]=0.59; CI 95% 0.41-0.85). Additionally, current MTH users presented lower median systolic blood pressure than women who have never used MTH and previous users (113 mmHg,118,5 mmHg and 120 mmHg, respectively, (p=0.001).^15^ This study’s conclusions, while cautious, contrast with recent larger longitudinal assessments from cohort studies and clinical trials. In this case, the study’s cross-sectional nature may represent a limitation. The “timing hypothesis” not possible to assess in this study design could be determinant for a different outcome. While cross-sectional analyses constitute a substantial body of evidence generators, longitudinal assessments and the “magic of randomization” may be informative for certain research questions.
